# Short-term effects of noisy pressure support ventilation in patients with acute hypoxemic respiratory failure

**DOI:** 10.1186/cc13091

**Published:** 2013-10-31

**Authors:** Peter M Spieth, Andreas Güldner, Robert Huhle, Alessandro Beda, Thomas Bluth, Dierk Schreiter, Max Ragaller, Birgit Gottschlich, Thomas Kiss, Samir Jaber, Paolo Pelosi, Thea Koch, Marcelo Gama de Abreu

**Affiliations:** 1 Pulmonary Engineering Group, Department of Anesthesiology and Intensive Care Medicine, University Hospital Dresden, Fetscherstrasse 74, 01307 Dresden, Germany; 2 Department of Electronic Engineering, Federal University of Minas Gerais, Avenida Antonio Carlos 6627, Belo-Horizonte, MG, Brazil; 3 Department of Visceral, Thorax and Vascular Surgery, University Hospital Dresden, Fetscherstrasse 74, 01307 Dresden, Germany; 4 Department of Anesthesiology and Intensive Care Medicine, University of Montpellier, Avenue Augustin Fliche, 34295 Cedex 5, Montpellier, France; 5 Department of Surgical Sciences and Integrated Diagnostics, University of Genoa, Largo Rosanna Benzi 8, 16132 Genova, Italy

## Abstract

**Introduction:**

This study aims at comparing the very short-term effects of conventional and noisy (variable) pressure support ventilation (PSV) in mechanically ventilated patients with acute hypoxemic respiratory failure.

**Methods:**

Thirteen mechanically ventilated patients with acute hypoxemic respiratory failure were enrolled in this monocentric, randomized crossover study. Patients were mechanically ventilated with conventional and noisy PSV, for one hour each, in random sequence. Pressure support was titrated to reach tidal volumes approximately 8 mL/kg in both modes. The level of positive end-expiratory pressure and fraction of inspired oxygen were kept unchanged in both modes. The coefficient of variation of pressure support during noisy PSV was set at 30%. Gas exchange, hemodynamics, lung functional parameters, distribution of ventilation by electrical impedance tomography, breathing patterns and patient-ventilator synchrony were analyzed.

**Results:**

Noisy PSV was not associated with any adverse event, and was well tolerated by all patients. Gas exchange, hemodynamics, respiratory mechanics and spatial distribution of ventilation did not differ significantly between conventional and noisy PSV. Noisy PSV increased the variability of tidal volume (24.4 ± 7.8% vs. 13.7 ± 9.1%, *P* <0.05) and was associated with a reduced number of asynchrony events compared to conventional PSV (5 (0 to 15)/30 min vs. 10 (1 to 37)/30 min, *P* <0.05).

**Conclusions:**

In the very short term, noisy PSV proved safe and feasible in patients with acute hypoxemic respiratory failure. Compared to conventional PSV, noisy PSV increased the variability of tidal volumes, and was associated with improved patient-ventilator synchrony, at comparable levels of gas exchange.

**Trial registration:**

ClinicialTrials.gov, NCT00786292

## Introduction

Mechanical ventilation is often used as a lifesaving intervention to restore or maintain adequate oxygenation in critical ill patients. However, mechanical ventilation *per se* can induce or aggravate pulmonary damage, a condition known as ventilator-induced lung injury (VILI) [1]. It has been shown that early restitution of spontaneous breathing activity is associated with benefits regarding pulmonary function as well as decreased need for vasopressors or sedative drugs in patients with acute respiratory failure [2]. Early induction of spontaneous breathing may lead to faster weaning of the patient from mechanical ventilation and therefore shorten the length of stay in the intensive care unit or in the hospital.

Pressure support ventilation (PSV) is one of the most common modes of assisted spontaneous breathing [3]. During PSV, spontaneous breaths are supported by a fixed pressure support, which may result in a respiratory pattern with relatively low tidal volume variability [4]. However, decreased variability of tidal volumes has been shown to be associated with impaired lung function and increased lung damage [5-7]. Experimental studies have demonstrated that the combination of assisted spontaneous breathing and variable tidal volumes by means of variable pressure support levels (variable pressure support ventilation - noisy PSV) may improve lung function and reduce pulmonary inflammatory response [8-10]. Interestingly, the ‘optimal’ level of pressure support variability is very close to the respiratory variability seen in healthy subjects [11,12].

Noisy PSV can increase the variability of the respiratory pattern even when it is intrinsically reduced, as often seen in critically ill patients [13,14]. However, noisy PSV has been tested only in experimental models of acute respiratory distress syndrome (ARDS). Thus, we designed this study to test the safety and feasibility of noisy PSV in patients with acute hypoxemic respiratory failure. We hypothesized that, in the short term, noisy PSV would result in comparable lung function and better patient comfort than conventional PSV, proving safe for clinical use.

## Material and methods

### Study design

This monocenter, randomized crossover study was approved by the institutional ethics review board of the University Hospital Dresden, Germany (EK 276112007) and registered at ClinicialTrials.gov (NCT00786292). Thirteen patients from the anesthesiological and surgical intensive care units of the University Hospital Dresden who were diagnosed with acute hypoxemic respiratory failure were enrolled in this study. Written informed consent was obtained from patient’s legal representative. Patients were included in the study if all of the following criteria were fulfilled: a) age between 18 and 75 years; b) ratio of arterial partial pressure of oxygen and inspired oxygen fraction (PaO_2_/F_I_O_2_) between 150 and 300 mmHg; total duration of mechanical less than 14 days; mechanical ventilation with biphasic positive airway pressure ventilation with at least 20% of minute ventilation corresponding to spontaneous breathing, or assisted mechanical ventilation with pressure support ventilation, at time of inclusion. Patients were excluded from the study if at least one of the following criteria was present: i) body mass index > 35; ii) esophageal disease; iii) neuromuscular disease; iv) instable thorax; v) pneumothorax; brain injury/increased intracranial pressure; vi) brain tumor; vii) agitation; viii) increased need for vasopressors or hemodynamic drug support, defined as dopamine or dobutamine dosage > 5 μg/kg/min, noradrenaline > 2 μg/kg/min, or use of vasopressin or milrinone; ix) chronic pulmonary disease; x) acute coronary insufficiency; xi) participation in other clinical trials in the preceding four weeks.

The study intervention was mechanical ventilation with conventional or variable PSV for one hour each in all patients, in a randomized fashion. One of two envelopes containing either conventional or variable PSV was drawn to determine the sequence of mechanical ventilation modes.

After preparing the measurement devices, patients were switched to the study ventilator and a stabilization period of 20 minutes was allowed prior to baseline measurements. Following baseline measurements, the sequence of conventional and noisy PSV was randomized and the patients were ventilated for one hour with each mode. The study was interrupted if one of the following criteria was fulfilled: 1) acute change of mental status; 2) diaphoresis; 3) exacerbated movement of intercostal muscles; 4) dyspnea; 5) paradoxical abdominal breathing; 6) respiratory rate >30/min or <6/min; g) arterial pH <7.30; 7) heart rate increase >20% compared to baseline, or <50/min, or >110/min (absolute); 8) mean arterial pressure (MAP) increase >20% compared to baseline, or <70 mmHg, or >110 mmHg (absolute).

After finishing the study protocol, patients were switched back to their initial ventilator and therapy was continued at the discretion of the treating physician.

### Monitoring and instrumentation

All patients were monitored with electrocardiography, pulse oxymetry and invasive arterial blood pressure measurement according to the current standard of the intensive care units. Additionally, an esophageal balloon catheter (Erich Jäger, Höchberg, Germany) and electrical impedance tomography (EIT Evaluation Kit II, Dräger Medical, Lübeck, Germany) were used for study specific measurements in case of the absence of contraindications to these procedures. Epidemiological data, current therapy and diagnosis were reviewed and captured using the intensive care units’ electronic patient data management system (ICM, Dräger Medical).

### Mechanical ventilation

All patients were already on assisted spontaneous breathing prior to the start of the study, and laid in the supine and semirecumbent 45° position. A commercial intensive care ventilator (Dräger Evita XL, Dräger Medical) was used for both PSV and noisy PSV. To perform noisy PSV, the ventilator was remote controlled by an external computer using the Dräger MediBus protocol as previously described [10]. During noisy PSV, pressure support values were generated randomly and followed a Gaussian (normal) distribution, whereby the coefficient of variation (100 × standard deviation (SD)/mean value) was 30%, as described in detail in a previous study from our group [12]. The ventilator settings and alarm limits are defined in Table 1. Briefly, pressure support was titrated to reach tidal volumes (V_T_) of approximately 8 ml/kg ideal body weight. We chose that level since it represents the upper limit of V_T_ still compatible with protective ventilation. The level of positive end-expiratory pressure (PEEP) and fraction of inspired oxygen (F_I_O_2_) were kept unchanged according the current therapy. The flow trigger was set at 3 L/min and cycling-off criteria at 25% of peak inspiratory flow in all patients, for both conventional and noisy PSV.

**Table 1:** Ventilator settings

***Settings***	***PSV***	***Noisy PSV***
**Respiratory rate**	Spontaneous	Spontaneous
**P**_**ASB **_**(driving pressure)**	Targeted at V_T_ ≈ 8 mL/kg	Targeted at V_T_ ≈ 8 mL/kg
**PEEP**	According to current therapy	According to current therapy
**Ramp**	0.20	0.20
**F**_**I**_**O**_**2**_	According to current therapy	According to current therapy
**Flow trigger**	3 L/min	3 L/min
**Coefficient of variation of pressure support**	0%	30% (normal distribution of randomly generated values)
**Alarm limits**		
Peak airway pressure	35 cm H_2_O	35 cm H_2_O
Minute ventilation	± 50% of current therapy	± 50% of current therapy
Respiratory rate (lower)	6	6
Respiratory rate (higher)	30	30

PSV, pressure support ventilation; V_T_, tidal volume; PEEP, positive end-expiratory pressure; F_I_O_2_, inspiratory oxygen fraction.

### Measurements

Gas exchange variables (PaO_2_/F_I_O_2_; arterial partial pressure of carbon dioxide (PaCO_2_)) were measured by point of care blood gas analysis (ABL800, Radiometer, Copenhagen, Denmark). Hemodynamic variables (heart rate (HR), MAP) were recorded from the hemodynamic monitoring system. Airway flow signals were continuously recorded from the mechanical ventilator at a sample frequency of 125 Hz using the MediBus interface to calculate V_T_ by means of numerical integration of the flow signal over time. Airway (peak and mean airway pressures (P_aw_ peak, P_aw_ mean)) and esophageal pressures were measured, digitized and recorded using previously described routines [15]. EIT was used to measure relative changes in thoracic impedance as a surrogate for the spatial distribution of ventilation. EIT data was recorded and stored in the device and processed offline using a routine developed by our group. The transpulmonary pressure (P_L_) was calculated as the difference between airway and esophageal pressure. Pressure time product (PTP) was calculated by numerical integration of esophageal pressure and inspiratory time. The coefficient of variation of V_T_ (CV V_T_) was calculated as the ratio of the standard deviation and the mean of V_T_. Patient-ventilator asynchrony was assessed from respiratory signals and quantified as described elsewhere [16]. Briefly, we computed the occurrence of ineffective triggering (single patient effort fails to trigger the ventilator and hence no pressure support is delivered), double triggering (ventilator triggered twice without an expiration between cycles), as well as double inspiratory effort (patient had two inspiratory efforts, just one effort triggered the ventilator), and values were added to obtain the total number of asynchrony events.

### Statistical analysis

Values are given as mean and SD unless stated otherwise. Differences between noisy and conventional PSV were tested using paired *t* tests, or Wilcoxon signed-rank test, as appropriate. EIT data were compared using repeated measures two-way analysis of variance (ANOVA) (factors: group and region) with Bonferroni adjustment. All tests were performed using GraphPad Prism (Version 5a, GraphPad Software, La Jolla, CA, USA). Statistical significance was accepted at *P* <0.05.

## Results

**Figure 1:**
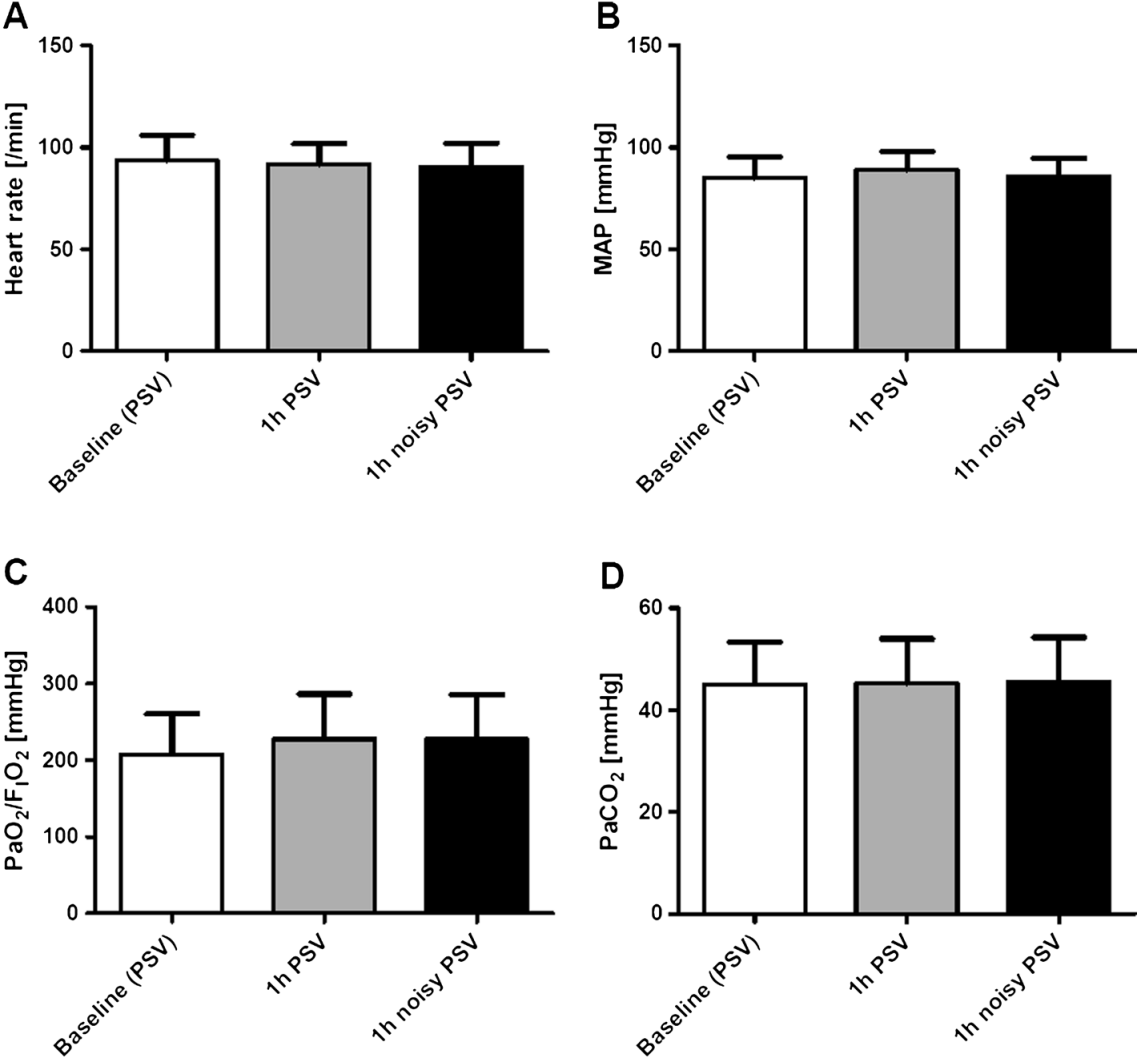
**Hemodynamics and gas exchange.** Hemodynamics and gas exchange during (conventional) pressure support ventilation (PSV) and noisy (variable) PSV. **(A)** Heart rate (HR); **(B)** mean arterial blood pressure (MAP); **(C)**, ratio of arterial partial pressure of oxygen and inspired oxygen fraction (PaO_2_/F_I_O_2_); **(D)** arterial partial pressure of carbon dioxide (PaCO_2_).

We enrolled eleven male and two female patients, whose demographic and clinical data are summarized in Table 2. Both, PSV and noisy PSV were feasible and could be safely applied in all patients. We did not find any signs of distress or discomfort associated with mechanical ventilation in this study. Furthermore, we could not identify any negative effects of noisy PSV on gas exchange, hemodynamics or respiratory variables associated with the application of variable pressure support levels. Accordingly, the interruption criteria were not fulfilled during conventional or noisy PSV. Major variables of hemodynamics (HR, MAP) and gas exchange (PaO_2_/F_I_O_2_, PaCO_2_) (Figure 1A-C), as well as acid/base status (pH, HCO_3_^-^, base excess (BE), lactate) (Table 3) did not differ significantly between PSV and noisy PSV.

**Table 2:** Demographics

***Patient number***	***Age***	***IBW (kg)***	***Duration of ventilation***	***Airway***	***PEEP***	***F***_***I***_ ***O***_***2***_	***Sedative***	***Analgetic***	***PaO***_***2***_ ***/F***_***I***_ ***O***_***2 ***_ ***at inclusion***	***Ramsay score***	***APACHE II score***	***Primary diagnosis***
**1**	55	66	9	TC	6	0.45	None	Sufentanil	228	4	12	Acute bowel ischemia
**2**	48	70	2	ETT	10	0.40	Propofol	Sufentanil	217	4	3	Acute peritonitis
**3**	22	48	3	ETT	12	0.50	Midazolam	Sufentanil	157	4	7	Pneumonia
**4**	56	68	4	ETT	10	0.40	None	Sufentanil	279	2	3	Pneumonia
**5**	41	57	5	ETT	10	0.40	Midazolam	Sufentanil	240	4	9	Sepsis
**6**	19	75	14	TC	10	0.45	Clonidin	Sufentanil	240	2	4	Lung contusion
**7**	72	78	6	ETT	10	0.45	None	Sufentanil	298	4	7	Perforated aortic aneurysm
**8**	69	75	11	TC	10	0.40	None	Sufentanil	165	2	7	Pneumonia
**9**	74	71	9	ETT	10	0.80	None	Sufentanil	165	3	7	Perforated aortic aneurysm
**10**	34	75	10	TC	10	0.45	Clonidin	Sufentanil	221	3	2	Lung contusion
**11**	18	82	8	ETT	12	0.40	Midazolam	Sufentanil	188	4	10	Pneumonia
**12**	73	71	5	ETT	12	0.40	None	Sufentanil	203	5	9	Lung contusion
**13**	60	70	2	ETT	8	0.40	Propofol	Sufentanil	248	2	7	Anastomotic insufficiency
**Summary counts or mean and min-max**	49.3 (18-74)	69.7 (48-82)	6.8 (2-14)	4 × TC	10 (6-12)	0.45 (0.40-0.80)	6 × none	13 × sufentanil	219 (157-298)	3.3 (2-5)	6.7 (2-12)	7 × direct
				9 × ETT			3 × midazolam					6 × indirect
							2 × propofol					
							2 × clonidin					

IBW, ideal body weight; PEEP, positive end-expiratory pressure; FIO_2_, fraction of inspired oxygen; *PaO*_*2*_, arterial partial pressure of oxygen; APACHE II, acute physiology and chronic health evaluation. TC, tracheal cannula; ETT, endotracheal tube.

**Table 3:** Blood and respiratory variables

	***Baseline (PSV)***	***PSV***	***Noisy PSV***
**pH**	7.41 ± 0.13	7.41 ± 0.05	7.41 ± 0.05
**HCO**_**3**_^**- **^**(mmol/L)**	27.1 ± 4.0	27.1 ± 3.8	27.2 ± 3.8
**BE**	3.0 ± 3.9	2.9 ± 3.8	3.0 ± 3.8
**Lactate (mmol/L)**	1.2 ±0.4	1.0 ± 0.3	1.1 ±0.3
**Lowest V**_**T**_**/kg IBW (mL/kg)**	4.6 ± 2.3	5.3 ± 2.3	3.0 ± 1.6**
**Mean V**_**T**_**/kg IBW (mL/kg)**	9.0 ± 1.8	9.0 ± 1.3	8.7 ± 1.3
**Highest V**_**T**_**/kg IBW (mL/kg)**	13.1 ± 5.8	12.6 ± 3.4	14.3 ± 4.0*
**RR (/min)**	18.8 ± 6.0	18.9 ± 7.0	19.0 ± 6.4
**T**_**i**_**/T**_**tot**_	0.3 ± 0.1	0.3 ± 0.1	0.3 ± 0.1

Values are mean ± standard deviation (SD). PSV, pressure support ventilation; BE, base excess; V_T_/kg IBW, tidal volume per kg of ideal body weight; RR, respiratory rate, T_I_/T_tot_, ratio of inspiratory time to total breath duration. Statistical significance according to Wilcoxon paired, two-sided signed rank test between PSV and noisy PSV at *P* <0.05. *, *P* <0.05; **, *P* <0.01.

**Figure 2:**
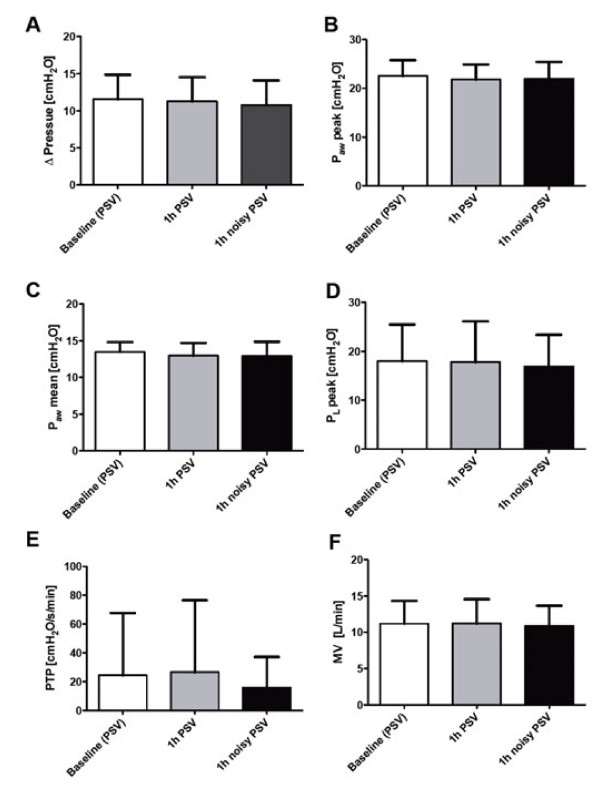
**Respiratory variables.** Respiratory variables during (conventional) pressure support ventilation (PSV) and noisy (variable) PSV. **(A)** Mean pressure support (Δ pressure); **(B)** peak airway pressure (P_aw_ peak); **(C)** mean airway pressure (P_aw_ mean); **(D)** peak transpulmonary pressure (P_L_ peak); **(E)** pressure time product (PTP); **(F)** minute ventilation (MV).

**Figure 3:**
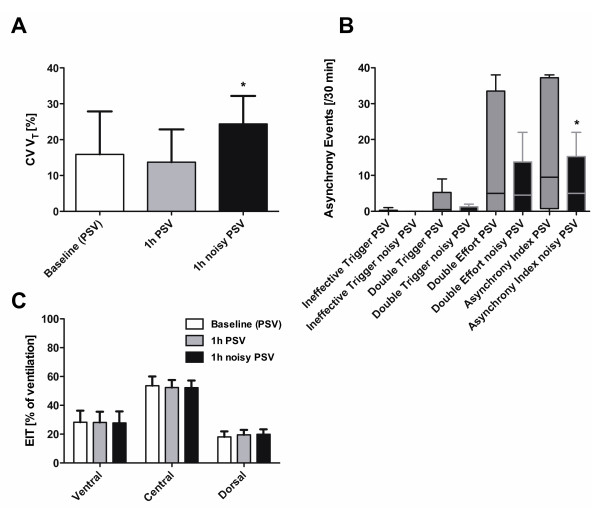
**Variability, patient-ventilator asynchrony and regional distribution of ventilation.** Variability, patient-ventilator asynchrony, and distribution of regional ventilation by electrical impedance tomography (EIT) during (conventional) pressure support ventilation (PSV) and noisy (variable) PSV. **(A)** coefficient of variation of tidal volume (CV V_T_); **(B)** asynchrony events; **(C)** percentage of ventilation assessed by electrical impedance tomography (EIT); *, *P* <0.05 vs. PSV. Data in panel **A** and **C** are presented as mean and standard deviation (SD), data in panel **B** as median and range. Two-way analysis of variance (ANOVA), paired *t* tests and Wilcoxon signed-rank tests were used as appropriate.

Pressure support levels (Δ pressure) and airway pressures (peak, mean and transpulmonary pressure) as well as pressure time product (PTP) and minute ventilation did not differ significantly between groups (Figure 2A-F). CV V_T_ was significantly higher with noisy PSV, as compared to conventional PSV (Figure 3A). During noisy PSV, the lowest V_T_ values were lower, while the highest V_T_ values were higher than during conventional PSV (Table 3). Accordingly, the range of V_T_ values during noisy PSV (11.3 ± 4.9 mL/kg) was higher than during conventional PSV (7.3 ± 5.0 mL/kg, *P* < 0.01).

Noisy PSV was associated with a decreased number of asynchrony events compared to conventional PSV (Figure 3B).

Mean values of V_T_, mean respiratory rate (RR) and mean ratio of inspiratory time to total time of the breathing cycle (T_i_/T_tot_) did not differ significantly between assisted ventilation modes (Table 3). Also, the regional distribution of ventilation was comparable during noisy and conventional PSV (Figure 3C).

## Discussion

The main findings of the present study were that, in a mixed population of patients with acute hypoxemic respiratory failure: 1) noisy PSV was not associated with adverse events; 2) gas exchange, hemodynamics, mean respiratory variables and regional distribution of ventilation did not differ significantly between conventional and noisy PSV; 3) noisy PSV was associated with significantly higher tidal volume variability than conventional PSV; 4) noisy PSV was associated with improved patient-ventilator synchrony compared to conventional PSV.

To the best of our knowledge, this is the first clinical trial on noisy PSV in patients. Since this was a safety and feasibility study, we focused on patients with mild to moderate acute hypoxemic respiratory failure, who were already mechanically ventilated with conventional PSV and had no major comorbidities. Also, we performed noisy PSV for a very short period of time in order to minimize potential risks. It is worth noting that none of the patients disclosed any sign of discomfort associated with the application of noisy PSV. Accordingly, no major changes in hemodynamics were observed during noisy PSV, suggesting that the new mode of assisted mechanical ventilation was well tolerated. Furthermore, noisy PSV was well accepted by the nursing and medical personnel.

The use of assisted mechanical ventilation in acute respiratory failure has been intensively debated in recent years. Papazian and colleagues [17] showed that the use of neuromuscular blocking agents and consecutive controlled mechanical ventilation within the first 48 h could reduce mortality of patients with severe, but not mild or moderate ARDS. In contrast, the risk of muscle dysfunction, for example rapid disuse atrophy of the diaphragm, has been reported even after short periods of controlled mechanical ventilation [18]. Also, it has been demonstrated that the induction of spontaneous breathing even in the early phase of ARDS can be beneficial in terms of reduced use of sedatives, vasopressors and reduced time on mechanical ventilation [19]. Those discrepancies may be explained by distinct transpulmonary pressures during different modes of mechanical ventilation.

It is worth noting that the transpulmonary pressure, which is the driving force leading to re-opening of collapsed lung regions, can be higher during assisted than controlled mechanical ventilation, especially in gravitationally dependent lung regions [19]. Since during ARDS the transpulmonary pressure gradient is increased along the ventral-dorsal axis [20], such effect may play a pivotal role for recruitment of dependent zones. On the other hand, excessive transpulmonary pressures may contribute to ventilator-associated lung injury [21]. In our patients, the mean transpulmonary pressures were comparable between conventional and noisy PSV, suggesting that the new mode of assisted mechanical ventilation did not pose this population at higher risk of lung injury.

It is well known that intrinsic variability in many organ systems is decreased during disease and the aging process [14,22]. However, it is not known whether the restoration of variability of organ rhythm, for example of V_T_ in mechanically ventilated patients, is beneficial. A growing body of laboratory evidence suggests that variable mechanical ventilation modes aiming at imitating physiological respiratory variability improve gas exchange and respiratory system mechanics, and reduce ventilator-associated lung injury [5,6,9]. Interestingly, the type of variability, that is natural (recorded from healthy subjects) or random (generated randomly by a computer), seems not to play a major role for the beneficial effects of variable ventilation [23]. On the other hand, observational clinical studies suggested that increased variability of respiratory variables is associated with faster weaning from mechanical ventilation [24]. Even though our patients showed a considerable amount of variability in respiratory variables during conventional PSV, such variability, as measured by the coefficient of variation, roughly doubled during noisy PSV, reaching values described for healthy subjects [11].

Since the level of pressure support largely determines V_T_[10], the finding that during noisy PSV the lowest V_T_ values were lower, while the highest V_T_ values were higher than during conventional PSV is not unexpected. Although the highest V_T_ values during noisy PSV is outside the protective range of 4 to 8 mL/kg, they occur only rarely, due to the Gaussian distribution of the pattern underlying the new mode of mechanical ventilation. Furthermore, experimental evidence has shown that variable ventilation with extreme V_T_ as high as 16 mL/kg and as low as 1.6 mL/kg occurring once every 20 to 30 minutes is associated with decreased ventilator lung histologic damage compared to non-variable ventilation with constant V_T_ of 6 mL/kg [5]. In addition, one should keep in mind that during noisy PSV, V_T_ values more protective than during conventional PSV also occurred in our population.

Different modes of assisted mechanical ventilation may lead to increased variability of respiratory variables in the clinical practice, most notably proportional assist ventilation (PAV) and neurally adjusted ventilator assist (NAVA). However, in contrast to PAV and NAVA, noisy PSV is able to restore the breath-by-breath variability of the respiratory pattern independent of a patient’s intrinsic respiratory variability. Thus, noisy PSV may increase the variability of the breathing pattern even in patients with reduced intrinsic variability, for example due to sedation or impairment of the respiratory center. Furthermore, noisy PSV does not require closed-loop mechanisms, rendering it technically simpler to implement and easier to handle compared to PAV and NAVA. Also, noisy PSV does not require insertion of esophageal catheters, making it attractive for patients with a contraindication for such catheters, and is devoid of movement artifacts.

In experimental models of the ARDS, noisy PSV led to an important improvement in arterial oxygenation compared to PSV [9,10,25]. In contrast, in our patients, the PaO_2_ did not differ significantly between conventional and noisy PSV. There are several possible explanations for the lack of improvement in gas exchange: 1) the time period under noisy PSV was relatively short; 2) our patients had less severe hypoxemic respiratory failure than previous models of ARDS; 3) the hypoxic pulmonary vasoconstriction was blunted. In experimental models of ARDS, redistribution of pulmonary blood flow toward better aerated lung tissue has been observed during noisy PSV [10], but may have been impaired in our patients, who were investigated later in the course of respiratory failure.

The distribution of regional ventilation in our patients did not differ significantly during PSV and noisy PSV. This finding is in agreement with previous laboratory data of our group showing that noisy PSV does not result in recruitment of dependent lung zones [8,10]. However, it must be kept in mind that since EIT is a cross-sectional technique, the present results cannot be extrapolated to the whole lungs.

Even though the total number of asynchrony events was relatively low during both modes of assisted mechanical ventilation, it is somewhat surprising that patient-ventilator synchrony was higher during noisy compared to conventional PSV. Improved synchrony has been well documented during assisted mechanical ventilation modes that apply pressure support proportionally to the inspiratory effort, like PAV and NAVA [26,27]. However, during noisy PSV, pressure support is dissociated from the inspiratory effort, as shown by our group in experimental ARDS [10]. A possible explanation is that the variation of pressure support covered much of the patients’ needs in terms of fluctuations of tidal volumes, and that patients adapt rapidly to breath-by-breath changes in pressure support. Accordingly, it could be speculated that part of the patient-ventilator asynchrony with conventional modes of assisted mechanical ventilation, most notably conventional PSV, originates in their inability to achieve higher variable respiratory patterns. Certainly, this issue deserves further investigation.

### Limitations

This study has several limitations. First, it was designed as a randomized crossover study aimed at testing the safety and feasibility, as well as physiological effects, of the newly developed ventilator mode noisy PSV. Therefore, we did not obtain long-term information or clinically relevant outcome variables. Second, since most of our patients were classified as moderate acute hypoxemic respiratory failure, the effects of noisy PSV in severe or mild as well as lung-healthy patients remain to be determined. Third, we investigated a selected population of patients, mainly in the postoperative period, and our results cannot be extrapolated to other situations. Fourth, we did not compare noisy PSV with other modes capable of increasing the variability of the breathing pattern, namely NAVA and PAV. Fifth, noisy PSV was performed with a coefficient of variation of 30% in pressure support in all patients, which resulted in a similar coefficient of variation in V_T_. Thus, possible individual needs may have been overlooked. However, such coefficient of variation has been shown to deliver the best compromise between gas exchange and lung mechanics in a model of ARDS [12]. Sixth, patient-ventilator asynchrony events have been registered from respiratory signals, including esophageal pressure. Thus, we were not able to quantify the asynchrony between the electric activation of the diaphragm and pressure support cycling-in and cycling-off. Furthermore, the counts of events could not be made in a blinded fashion, since tracings from noisy PSV and PSV differ considerably, and the mechanisms for such findings could not be addressed. Thus, our findings are indicative of an association.

## Conclusions

Noisy PSV proved safe and feasible in a population of patients with mild to moderate acute hypoxemic respiratory failure in the short term. Compared to conventional PSV, noisy PSV increased the variability of tidal volumes, and was associated with improved patient-ventilator synchrony, at comparable levels of gas exchange.

## Key messages

• Noisy PSV proved safe and feasible in patients with hypoxemic acute respiratory failure

• Noisy PSV increased the variability of tidal volumes compared to conventional PSV

• Noisy PSV was associated with better patient-ventilator synchrony than conventional PSV

## Abbreviations

ANOVA: Analysis of variance; ARDS: Acute respiratory distress syndrome; BE: Base excess; CV VT: Coefficient of variation of tidal volume; EIT: Electrical impedance tomography; FIO2: Fraction of inspired oxygen; HR: Heart rate; MAP: Mean arterial pressure; NAVA: Neurally adjusted ventilatory assist; PaCO2: Arterial partial pressure of carbon dioxide; PaO2/FIO2: Ratio of arterial partial pressure of oxygen and fraction of inspired oxygen; PAV: Proportional assist ventilation; Paw mean: Mean airway pressure; Paw peak: Peak airway pressure; PEEP: Positive end-expiratory pressure; PL: Transpulmonary pressure; PSV: Pressure support ventilation; PTP: Pressure time product; RR: Respiratory rate; SD: Standard deviation; Ti/Ttot: Ratio of inspiratory time to total time of the breathing cycle; VILI: Ventilator-induced lung injury; VT: Tidal volume.

## Competing interests

Drs. Gama de Abreu, Spieth and Koch were granted a patent on the variable pressure support ventilation mode of assisted ventilation (noisy PSV), which has been licensed to Dräger Medical AG (Lübeck, Germany).

## Authors’ contributions

PMS contributed to the study design, data acquisition, data analysis, and to drafting the manuscript. AG contributed to the data acquisition, to revising the manuscript, and to drafting the manuscript. RH contributed to the data acquisition, data analysis, and to revising the manuscript. AB contributed to the data acquisition, data analysis, and to revising the manuscript. TB contributed to the data acquisition, data analysis, and to revising the manuscript. DS contributed to the data acquisition, data analysis, and to revising the manuscript. MR contributed to the data acquisition, data analysis, and to revising the manuscript. BG contributed to the data acquisition, data analysis, and to revising the manuscript. TK contributed to the data acquisition, data analysis, and to revising the manuscript. SJ contributed to the study design, and to revising the manuscript. PP contributed to the study design, and to revising the manuscript. TK contributed to the study design, and to revising the manuscript. MGA contributed to the study design, data acquisition, data analysis, and to drafting the manuscript. All authors read and approved the final manuscript.
